# Agonist anti-GITR monoclonal antibody and stereotactic radiation induce immune-mediated survival advantage in murine intracranial glioma

**DOI:** 10.1186/s40425-016-0132-2

**Published:** 2016-05-17

**Authors:** Mira A. Patel, Jennifer E. Kim, Debebe Theodros, Ada Tam, Esteban Velarde, Christina M. Kochel, Brian Francica, Thomas R. Nirschl, Ali Ghasemzadeh, Dimitrios Mathios, Sarah Harris-Bookman, Christopher C. Jackson, Christina Jackson, Xiaobu Ye, Phuoc T. Tran, Betty Tyler, Vladimir Coric, Mark Selby, Henry Brem, Charles G. Drake, Drew M. Pardoll, Michael Lim

**Affiliations:** The Johns Hopkins University School of Medicine, Baltimore, USA; Department of Oncology, Baltimore, USA; Department Radiation Oncology, Baltimore, USA; Department of Neurosurgery, The Johns Hopkins University School of Medicine, 600 N. Wolfe St. Phipps Building Rm 123, Baltimore, 21287 MD USA; Bristol-Myers Squibb Company, San Francisco, CA USA; and the Brady Urological Institute, Baltimore, USA

**Keywords:** GITR, Immune checkpoint, Immunotherapy, Radiation, Gioblastoma, Antibody

## Abstract

**Background:**

Glioblastoma (GBM) is a poorly immunogenic neoplasm treated with focused radiation. Immunotherapy has demonstrated synergistic survival effects with stereotactic radiosurgery (SRS) in murine GBM. GITR is a co-stimulatory molecule expressed constitutively on regulatory T-cells and by effector T-cells upon activation. We tested the hypothesis that anti-GITR monoclonal antibody (mAb) and SRS together would confer an immune-mediated survival benefit in glioma using the orthotopic GL261 glioma model.

**Methods:**

Mice received SRS and anti-GITR 10 days after implantation. The anti-GITR mAbs tested were formatted as mouse IgG1 D265A (anti-GITR (1)) and IgG2a (anti-GITR (2a)) isotypes. Mice were randomized to four treatment groups: (1) control; (2) SRS; (3) anti-GITR; (4) anti-GITR/SRS. SRS was delivered to the tumor in one fraction, and mice were treated with mAb thrice. Mice were euthanized on day 21 to analyze the immunologic profile of tumor, spleen, and tumor draining lymph nodes.

**Results:**

Anti-GITR (1)/SRS significantly improved survival over either treatment alone (*p* < .0001) with a cure rate of 24 % *versus* 0 % in a T-lymphocyte-dependent manner. There was elevated intratumoral CD4+ effector cell infiltration relative to Treg infiltration in mice treated with anti-GITR (1)/SRS, as well as significantly elevated IFNγ and IL-2 production by CD4+ T-cells and elevated IFNγ and TNFα production by CD8+ T-cells. There was increased mRNA expression of M1 markers and decreased expression of M2 markers in tumor infiltrating mononuclear cells. The anti-GITR (2a)/SRS combination did not improve survival, induce tumor regression, or result in Treg depletion.

**Conclusions:**

These findings provide preclinical evidence for the use of anti-GITR (1) non-depleting antibodies in combination with SRS in GBM.

**Electronic supplementary material:**

The online version of this article (doi:10.1186/s40425-016-0132-2) contains supplementary material, which is available to authorized users.

## Background

Glioblastoma (GBM) is the most prevalent primary brain tumor in adults, with a bleak median survival of 1–2 years and a <10 % 5-year survival rate [1–3]. The current standard of care for GBM includes surgical resection, temozolomide chemotherapy, and radiation for recurrence, but with only modest improvements in survival the search has intensified for more effective therapies [4]. Preclinical studies have demonstrated early successes for the use of immunotherapy against glioma, including antibody-mediated blockade of checkpoints including anti-programmed death-1 (anti-PD-1), anti-cytotoxic T lymphocyte antigen-4 (anti-CTLA-4), and anti-4-1BB, particularly in combination with radiotherapy [5, 6]. As a result, clinical trials are now underway testing checkpoint inhibitors in human patients with GBM (NCT02017717).

Glucocorticoid-induced tumor necrosis factor related protein (GITR) is an immune checkpoint that belongs to the tumor necrosis factor receptor (TNFR) family and is expressed on T lymphocytes, natural killer cells, and granulocytes [7]. The GITR ligand (GITR-L) is expressed on antigen presenting cells (APCs) such as dendritic cells, macrophages, and B-cells [8]. While expressed at low basal levels in CD4+ and CD8+ effector T cells, GITR is upregulated 24–72 h following an antigenic stimulus and remains expressed at high levels for several days [8, 9]. Conversely, GITR is constitutively expressed in regulatory T cells (Tregs). It has been observed that binding of GITR in CD4+ and CD8+ T cells by its ligand results in increased effector function, cell proliferation, and possible resistance to Treg immunosuppression, whereas GITR stimulation in Tregs leads to FoxP3 loss, Treg instability, and a decline in suppressive function [10–12]. Anti-GITR monoclonal antibody (mAb) therapy has resulted in tumor regression in a number of tumor models, but has not yet been tested against murine glioma [12–17]. Similar to blockade of checkpoints PD-1 and lymphocyte activating gene (LAG)-3, among others, activation of TNFR family checkpoint molecules has not led to severe toxicities in preclinical models, making GITR an attractive target for future immune checkpoint modulation [18, 19].

Cells damaged by ionizing radiation up-regulate pro-inflammatory ligands and release cytokines and antigens that become immunogenic substrates for infiltrating immune cell activation [20, 21]. Stereotactic radiosurgery (SRS) has the advantage of delivering a high dose of radiation to the target while minimizing damage to surrounding tissues. Commonly used in the treatment of brain cancer, SRS can be an effective measure to treat microscopic residual disease and delay recurrence [22]. Studies of focal radiation in combination with checkpoint modulation in preclinical glioma and breast cancer models have exhibited favorable immune-mediated anti-tumor responses, and phase I/II clinical studies of radiation therapy combined with CTLA-4 blockade in prostate cancer have yielded encouraging outcomes [5, 6, 23, 24]. Here, we tested an anti-GITR IgG1 D265A (anti-GITR (1)) agonist and an anti-GITR IgG2a (anti-GITR (2a)) depleting mAb in combination with SRS in a murine intracranial glioma model. We found that anti-GITR (1)/SRS significantly improves survival and delays tumor progression in a CD4+ T cell dependent fashion that involves intratumoral M1 macrophage polarization, whereas anti-GITR (2a)/SRS does not prolong survival or delay tumor progression.

## Results

### GITR activation and stereotactic radiosurgery together produced long-term survivors and tumor regression in murine intracranial GL261

**Fig. 1:**
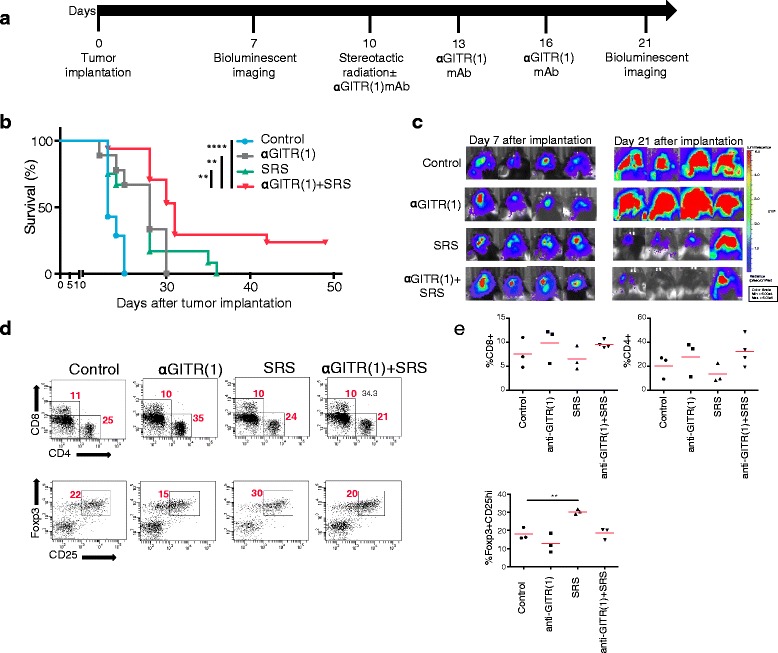
Eradication of intracranial GL261 tumors with anti-GITR (1) mAb plus SRS combination therapy. C57/BL6 mice were intracranially inoculated with 1.3 × 10^5^ GL261-luc cells, and after tumor establishment was confirmed by bioluminescence, mice were randomized into four groups of 10 mice per arm on day 7. Mice were administered focal radiation of 10 Gy 10 days after tumor implantation and/or received 200 μl anti-GITR (1) (10 mg/kg) by i.p. injection on days 10, 13, and 16 **a**. Mice were followed for survival **b**; curve-adjacent asterisks compare indicated curve to control. Tumor size was followed with bioluminescent imaging **c**; four representative mice are shown. Mice were sacrificed on day 21, and tumor infiltrating CD4 and CD8 T cells (gated on CD3+ cells) and Tregs (gated on CD4+ cells) were isolated and analyzed by flow cytometry **d** and **e**. Symbol and horizontal bar (**e**) denote single mouse and average value, respectively. *P < .05, **P < .01, ****P < .0001

To determine the effectiveness of GITR activation in the setting of adjuvant focal radiation against established GL261-luc murine glioblastoma tumors, an anti-GITR (1) agonist mAb was dosed according to previous studies of anti-GITR (1) in mouse tumor models and combined with stereotactic radiosurgery (SRS) dosed in a single fraction (Fig. 1a). SRS was dosed prior to anti-GITR in order to create an inflammatory tumor microenvironment favorable to immune modulation. Timing of SRS and anti-GITR dosage was based upon previously established schedules that correlate with tumor growth characteristics. Notably, anti-GITR (1) mAb was engineered as an IgG1 isotype with a D265A mutation that is unable to bind to Fc receptors; thus, anti-GITR (1) may only mediate bivalent agonism of GITR without concern for target cell depletion via antibody-dependent cell-mediated cytotoxicity (ADCC). Our results indicate that GITR activation alone did not inhibit tumor growth and resulted in survival outcomes not statistically different from controls (median survival anti-GITR (1) vs. control, 23 vs. 28 days) (Fig. 1b-c). Consistent with previous findings, SRS alone produced only partial tumor regression and no long-term survivors (median survival: 28 days) (Fig. 1b-c). By contrast, the combination of anti-GITR (1) mAb and SRS synergized to cure a subset of mice and produce a long-term survivor rate of 24 % (*P* < .0001, median survival: 31 days) (Fig. 1b-c). To examine whether there were differences in the intratumoral lymphocytic phenotype between treatment groups that could account for differences in survival, tumor-infiltrating lymphocytes (TIL) were isolated from each group on day 21 and analyzed for various T cell populations. While the proportion of CD8+ and CD4+ TIL were elevated in the combination anti-GITR (1)/SRS group relative to the negative control, this result was not statistically significant. Moreover, the ratio of CD8+ to CD4+ T cells was not significantly different between groups (data not shown). The proportion of CD4 + FoxP3 + CD25^hi^ regulatory T cells (Treg) was similar between the combination treatment group and control, but was significantly elevated in mice that received only SRS. In all, anti-GITR (1)/SRS combination therapy induced regression of GL261 tumors and produced a subset of cured long-term survivors in a manner that did not alter the overall proportion of TIL populations compared to controls.

### The survival advantage conferred by anti-GITR (1)/SRS combination therapy is dependent upon CD4+ cells and may be dependent upon CD8+ cells

**Fig. 2:**
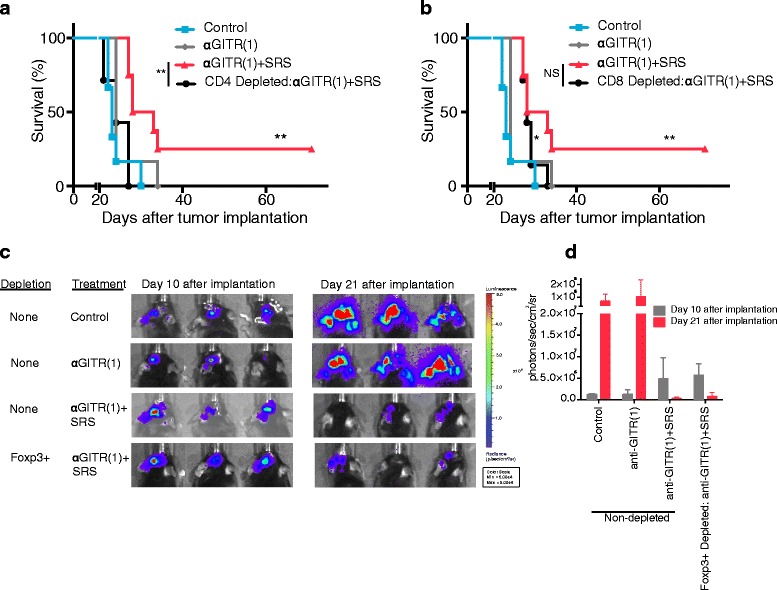
Survival benefit conferred by anti-GITR (1)/SRS treatment of murine glioma requires CD4+ T cells. C57/BL6 mice were inoculated with intracranial GL261-luc tumor, randomized to ≥7 mice per group and administered anti-GITR (1) and SRS as in Fig. 1. Mice were injected i.p. with 200 μl anti-CD4 (10 mg/kg) **a** and anti-CD8 (10 mg/kg) **b** depleting antibodies on days 5–7, 14, and 28 and followed for survival. Curve-adjacent asterisks compare indicated curve to control. The same control, anti-GITR (1), and anti-GITR (1)/SRS groups were used in (**a**) and (**b**) as the experiments were performed concurrently. The difference in survival between control mice and mice depleted of CD8+ T cells was statistically significant, but was not significant in the absence of CD4+ cells. FoxP3^DTR^ mice were inoculated intracranially with 1.3 × 10^5^ GL261-luc cells, randomized, injected i.p. with 200 μl of diphtheria toxin (50 ng/g) on day 8, 9 and every 2 days thereafter in order to achieve FoxP3+ cell depletion, administered anti-GITR (1) or SRS as in Fig. 1., and followed for tumor growth by bioluminescent imaging **c**. Tumor size was quantified by bioluminescence **d**. *P < .05; **P < .01. NS, non-significant

Immune subset analysis of TIL did not suggest statistically appreciable differences in the combination treatment group relative to the control. As such, T cell subset depletion studies were utilized to elucidate whether the mechanism of anti-tumor effect of combination treatment was dependent on a particular T cell population. Systemic antibody-mediated CD4+ T cell depletion abrogated the survival benefit conferred by anti-GITR (1)/SRS treatment, with a reduction in median survival of approximately 6 days (anti-GITR (1)/SRS CD4+ depleted vs. non-depleted, *P* < .01) (Fig. 2a). Of note, CD4+ depletion in control mice did not alter the rate of tumor progression relative to non-depleted control mice (data not shown). Systemic antibody-mediated CD8+ depletion resulted in eventual death of all depleted mice. However, CD8+ depletion did not abrogate the combination treatment-induced survival benefit, as demonstrated by a non-significant difference in median survival of CD8+ depleted mice compared to non-depleted mice, both treated with anti-GITR (1)/SRS (median survival 28 vs. 30 days, *P* > .05) (Fig. 2b). These data signify that while CD8+ T cells may not be integral to the combination treatment mechanism, the lack of long-term survivors in the CD8-depleted arm indicates that CD8+ cells are likely involved in the anti-tumor treatment effect.

To further explore which CD4+ T cell subset may have mediated the observed treatment effect, systemic depletion of FoxP3+ Tregs was achieved with the use of FoxP3^DTR^ transgenic mice and diphtheria toxin. These mice selectively express the diphtheria toxin receptor in regulatory T cells. Mice with intracranial GL261 treated with combination therapy exhibited similar tumor regression 21 days after tumor implantation in the Treg depleted and non-depleted groups (Fig. 2c-d). Our results indicate that depletion of FoxP3+ cells did not abrogate or apparently facilitate the anti-tumor effect of combination therapy as exhibited by bioluminescent imaging of the tumor. Together, these data suggest that the anti-tumor effect conferred by anti-GITR (1)/SRS treatment is dependent upon the CD4+ effector T cell population, may be dependent upon CD8+ T lymphocytes, and is not dependent upon FoxP3+ T cells.

### CD4+ and CD8+ TIL have elevated cytokine production in mice treated with anti-GITR (1)/SRS and a significantly elevated CD4 + IFNγ+/Treg ratio

**Fig. 3:**
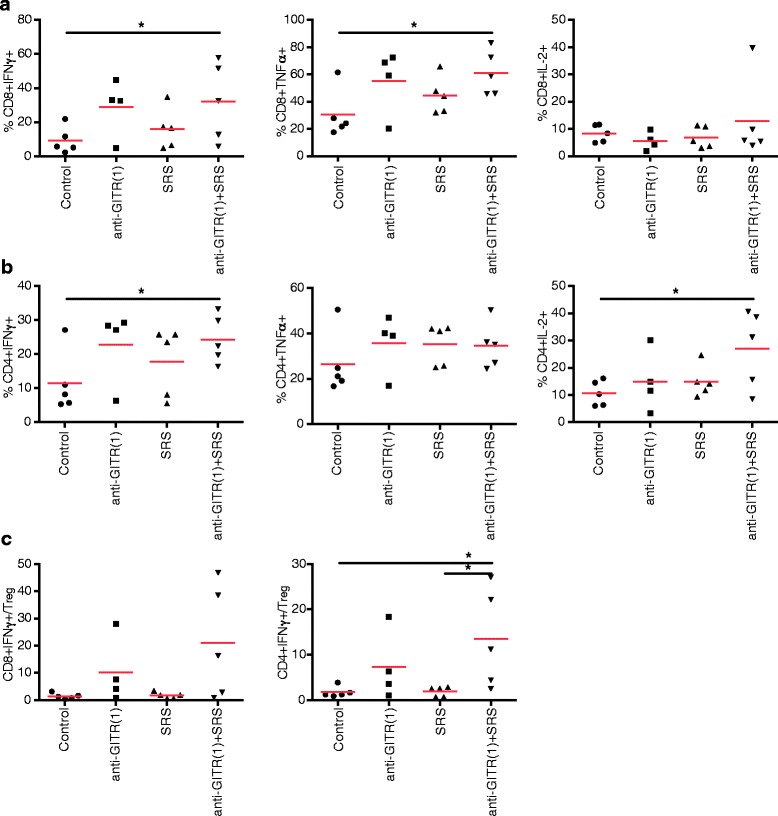
Tumor infiltrating lymphocytes (TIL) in the anti-GITR (1)/SRS group have a Th1 immunophenotype and elevated effector to Treg ratio. C57BL/6 mice were inoculated with GL261-luc tumor, randomized to groups of ≥5, and dosed with anti-GITR (1) and SRS as in Fig. 1. Mice were sacrificed on day 21, tumor infiltrating lymphocytes were isolated, cells were stimulated with PMA/Ionomycin, fixed, and permeabilized for staining of intracellular markers. **a** CD8+ and **b** CD4+ cell populations were analyzed by flow cytometry for expression of IFNγ, TNFα, and IL-2. Intratumoral effector CD8+ and CD4+ to Treg ratios in the anti-GITR (1)/SRS group were calculated **c**. Symbol and horizontal bar denote single mouse and average value, respectively. *P < .05

Given the apparent dependence of combination treatment on CD4+ T cells and possible dependence on CD8+ T cells, we sought to identify the effector lymphocyte phenotype potentially responsible for the treatment mechanism. Phenotypic analysis of TIL revealed significantly elevated percentage of CD4 + IFNγ + and CD8 + IFNγ + cells, CD8 + TNFα + cells, and CD4 + IL-2+ cells in mice treated with anti-GITR (1)/SRS relative to the control (*P* < .05 for all) (Fig. 3a-b). There were no significant differences in cytokine production between single-treatment arms and control. There was elevated IL-2 production by CD8+ T cells in the combination treatment group, but this result was not statistically significant. Given that Tregs are a significant source of immunosuppression in the tumor microenvironment, and that the proportion of the Treg subset among TIL in the combination treatment group was not diminished relative to the control (Fig. 1c), we hypothesized that the ratio of effector T cells to Tregs was elevated in the anti-GITR (1)/SRS group relative to the control. Such a relative increase in the pro-inflammatory phenotype could account for the ability of TIL receiving combination treatment to overcome local immunosuppression. Supporting this hypothesis, our results indicated a significantly elevated CD4 + IFNγ + to Treg ratio in mice receiving anti-GITR (1)/SRS relative to the control and SRS alone groups (*P* < .05), and a trend toward increased CD8 + IFNγ + to Treg ratio in the combination treatment group (Fig. 3c). These findings suggest that both CD4+ and CD8+ TIL may be involved in the anti-GITR (1)/SRS treatment regimen.

### Combination treatment yields intratumoral myeloid cells with overall lower expression of M2 and higher expression of M1 markers

**Fig. 4:**
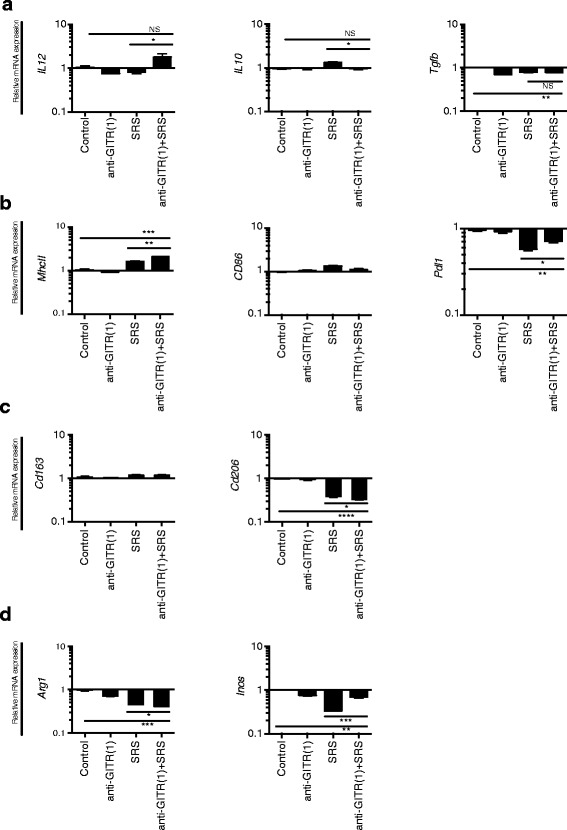
Anti-GITR (1)/SRS yields intratumoral myeloid cells with lower expression of M2 and higher expression of M1 markers. C57BL/6 mice were inoculated with GL261-luc tumor, randomized to groups of ≥5, and dosed with anti-GITR (1) and SRS as in Fig. 1. Mice were sacrificed on day 21, tumor infiltrating mononuclear cells were isolated, CD11b + CD45+ cells were sorted, and total RNA was isolated. Gene expression was calculated using real-time quantitative PCR analysis with *18 s* as the endogenous control. Column dot plots illustrate gene expression of **a** cytokines, **b** cell surface molecules, **c** cell surface receptors, and **d** cellular enzymes in each treatment group. *P < .05, **P < .01, ***P < .001, ****P < .0001

Our data indicate that Th1-type CD4+ cells may be an effector lymphocyte population in the combination treatment regimen. Th1 immune cells are pro-inflammatory and secrete IFNγ as their primary cytokine, among others. Given that Th1 CD4+ T cells are known to provide key activating signals to myeloid lineage cells, we hypothesized that the anti-GITR (1)/SRS treatment resulted in downstream activation of intratumoral resident and infiltrating myeloid-derived cells that ultimately contributed to the anti-tumor effect. To test this hypothesis, CD11b + CD45+ myeloid cells were isolated from tumor and queried for mRNA expression of M1 and M2 genetic markers via quantitative reverse transcriptase-PCR (Fig. 4, Additional file 1: Figure S1). Macrophages exist on a phenotypic continuum from the M1 (inflammatory and anti-tumorigenic) phenotype, to the M2 (regulatory and pro-tumorigenic) phenotype [25]. This population included microglia as well as tumor-infiltrating mononuclear cells. There was increased expression in the combination treatment group of M1 markers *IL12* (vs. SRS alone, *P* < .05; Fig. 4a) and *MhcII* (vs. SRS alone, *P* < .01; vs. control *P* < .001; Fig. 4b). Expression of M1 marker *Inos* was elevated in the combination treatment relative to SRS alone (*P* < .001) but was decreased relative to control (*P* < .01) (Fig. 4d). The M1 marker *Cd86* and M2 marker *Cd163* were not significantly different in the combination treatment group relative to SRS only or control (Fig. 4b-c). There was decreased expression in the combination treatment group of M2 markers *IL10* (vs. SRS alone, *P* < .05), *Cd206* (vs. SRS alone, *P* < .05; vs. control, *P* < .0001), and *Arg1* (vs. SRS alone, *P* < .05; vs. control, *P* < .001). Finally, expression of *Tgfb* (*P* < .01) and *Pdl1* (*P* < .01) was decreased in the combination treatment group relative to the control, although there was significantly higher expression of *Pdl1* in the anti-GITR (1)/SRS group relative to SRS alone (*P* < .05) (Fig. 4a-b). Taken together, these data suggest that the combination treatment induces up-regulation of genes involved in the pro-inflammatory M1 phenotype, with the exception of iNOS, and down-regulation of the phenotypically immunosuppressive M2 genes in resident microglia and tumor-infiltrating myeloid cells.

### The anti-GITR IgG 2a antibody/SRS combination does not prolong survival, does not induce intracranial GL261 tumor regression, and does not result in reduction of intracranial tumoral Tregs

**Fig. 5:**
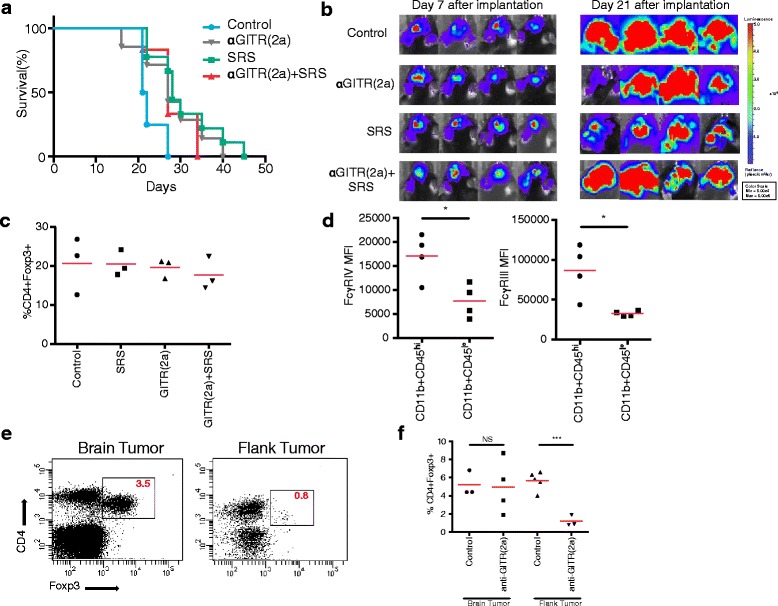
Lack of intracranial tumor eradication and Treg depletion with the anti-GITR IgG 2a antibody/SRS combination. C57BL/6 mice were intracranially inoculated with GL261-luc tumor, randomized to groups of ≥8, and dosed with 200 μl of anti-GITR (2a) (10 mg/kg) on day 10, 13, 16 and/or SRS (10 Gy) on day 10. Mice were followed for survival, **a** and tumor growth was assessed by bioluminescent imaging **b**. Mice were sacrificed on day 21, tumor infiltrating Tregs were isolated and analyzed by flow cytometry **c**. CD11b + CD45+ tumor resident microglia and tumor infiltrating mononuclear cells were isolated on day 21, analyzed by flow cytometry (gated on CD3+ cells), and mean fluorescence intensity (MFI) of FcγRIII and IV expression was calculated **d**. Flank tumors were established in C57BL/6 mice by subcutaneous inoculation of 2 × 10^6^ GL261-luc cells in a volume of 100 μl, and intracranial tumors were established as in Fig. 1. Mice were dosed i.p with 200 μl of anti-GITR (2a) (10 mg/kg) on days 10, 13, and 16, sacrificed on day 17, and tumor infiltrating lymphocytes were harvested and analyzed by flow cytometry for FoxP3 expression (gated on CD3+ cells) **e**-**f**. Symbol and horizontal bar denote single mouse and average value, respectively. *P < .05, ***P < .001. NS, non-significant

Because Tregs constitutively express GITR, we sought to test an anti-GITR antibody that induced cell death via antibody-dependent cell mediated cytotoxicity (ADCC). We predicted that the up-regulation of GITR on Tregs would result in disproportionate depletion of Tregs relative to CD4+ and CD8+ T cells after treatment with the anti-GITR IgG 2a (anti-GITR (2a)) mAb, as has been observed in flank tumor models [26]. We hypothesized that those mice treated with anti-GITR (2a) would exhibit significant intracranial GL261 tumor regression relative to controls as a result of reductions in intratumoral Treg numbers. To test this, a survival study identical to that in Fig. 1 was conducted using anti-GITR (2a) in place of anti-GITR (1) (Fig. 5). Notably, the anti-GITR (2a) mAb differs from anti-GITR (1) in the Fc region only. Surprisingly, anti-GITR (2a) in combination with SRS did not provide significant survival benefit over the control or either treatment alone (Fig. 5a). Indeed, bioluminescent imaging of tumors at day 21 after implantation revealed significant tumor growth in the combination treatment group comparative to that of the control group (Fig. 5b). In order to gain insight into the inability of the anti-GITR (2a)/SRS to produce survival benefit, TIL were analyzed at day 21 for the distribution of T cell populations across treatment groups. Anti-GITR (2a) alone or in combination with SRS did not induce significant intratumoral depletion of Tregs (Fig. 5c). It is known that anti-GITR (2a) relies upon the presence of activating Fcγ-receptors (FcγR) on antigen presenting cells (APCs) to mediate cell depletion via ADCC [26]. As such, we hypothesized that activating FcγR may be absent in the microglia, which are the resident APCs of the brain. Myeloid cells were harvested from intracranial GL261 tumors at day 21 and analyzed for expression of activating FcγRIII and FcγRIV (Fig. 5d). Supporting our hypothesis, the data suggest that intratumoral CD11b + CD45^lo^ resident microglia express significantly lower levels of activating FcγR relative to intratumoral CD11b + CD45^hi^ mononuclear cells, which may account for the inability of anti-GITR (2a) to induce Treg depletion through ADCC in brain tumor.

The efficacy of anti-GITR (2a) in Treg depletion may be greater in flank tumor models because of the increased presence of activating FcγR on peripheral mononuclear cells relative to microglia. We tested this hypothesis by simultaneously treating mice implanted with either intracranial or flank tumors with three doses of anti-GITR (2a), then harvesting tumors for flow cytometric analysis of Treg populations (Fig. 5e, f). While brain tumors treated with anti-GITR (2a) did not have significantly lower levels of intratumoral CD4 + FoxP3+ cells relative to control, anti-GITR (2a) treatment in flank tumors did result in a significantly diminished proportion of CD4 + FoxP3+ relative to control (Fig. 5e, f).

To summarize, these data signify that the anti-GITR (2a) antibody in combination with SRS does not provide a significant survival benefit in mice with intracranial GL261 relative to the control and cannot mediate intratumoral depletion of GITR-expressing cells, specifically Tregs, possibly owing to the relatively low expression of activating FcγR on intratumoral microglia.

## Discussion

The advent of immunotherapeutics has introduced new and exciting opportunities for the treatment of a variety of advanced cancers. It is important to bear in mind, however, that brain tumors such as GBM pose dual obstacles in the search for effective immunotherapies: low immunogenicity and residence in the immunologically distinct cranial vault [27]. In spite of this, pre-clinical models have shown promising efficacy of checkpoint inhibitors against glioma, and clinical trials of checkpoint blockade in human GBM are underway [5, 27].

Toxic side effects emerging from the blockade of the first generation of immune checkpoint targets have encouraged study of other checkpoint molecules with less severe autoimmune sequelae [28]. The TNFR family of checkpoint molecules including GITR, 4-1BB and OX40 is an attractive target for immunotherapy because of the relative lack of significant autoimmunity when stimulated [18, 19]. Stimulation of TNFR checkpoints 4-1BB and OX40 in pre-clinical glioma models has demonstrated immune-mediated regression of tumors with low resulting toxicity [5, 29, 30]. The effect of an anti-GITR agonist in the setting of murine glioma has not been investigated prior to this study, although GITR activation is an appealing strategy for its dual stimulatory influence on effector T cells and suppressive effect on Tregs [8, 10–13, 15, 17]. Here, we report that anti-GITR IgG1 agonist mAb in combination with SRS induces significant tumor regression and produces long-term survivors in murine intracranial glioma. These effects appear to be dependent upon CD4+ effector cells and may be dependent upon CD8+ cells. Additionally, we report the lack of efficacy of an anti-GITR IgG2a mAb alone or in combination with SRS to produce tumor regression or long-term survival in intracranial glioma, potentially owing to differences in Fc receptor expression by CNS-resident APCs.

We observed that anti-GITR (1)/SRS therapy significantly prolonged survival, whereas either therapy alone did not provide any survival benefit (Fig. 1a-b). Radiation has been shown to potentiate immune checkpoint blockade in intracranial and flank tumor models [5, 6, 23]. One hypothesis is that the damage of tumor cells produces an immunogenic substrate for infiltrating effector lymphocytes through the release of antigens as well as upregulation of cytokines and pro-inflammatory ligands [31–35]. In our experiments, however, initial TIL analysis did not demonstrate appreciable differences in the Treg or CD4+ and CD8+ populations between treatment groups (Fig. 1c). Indeed, our results support the hypothesis that the anti-tumor effect is more dependent upon differences in cytokine secretion between CD4+ and CD8+ cells rather than relative differences in tumor-infiltrating lymphocyte density (Fig. 3). Previous studies of anti-GITR treatment in flank tumor models have shown decreased intratumoral Treg infiltrate and elevated CD8+ relative to CD4 + Foxp3+ TIL, while studies of intracranial glioma treated with focal radiation with or without TNFR checkpoint stimulation also demonstrated elevated levels of CD4+ and CD8+ TIL [5, 6, 12, 14]. It is important to note that studies of anti-GITR in tumor models vary in the isotype of anti-GITR mAb used—those that treat with a rat IgG2b mAb would expect to see some T cell depletion as they share the isotype with a number of in vivo depleting mAbs (i.e., GK1.5, YTS191, YTS169, etc.), and those testing an anti-GITR mouse IgG2a should expect to see ADCC pathway cell depletion mediated by FcγR interactions [14, 26]. Our use of a mouse IgG1 anti-GITR mAb may explain the lack of reduction in CD4 + FoxP3+ cells, but does not necessarily account for the lack of elevated CD4+ and CD8+ effector cell infiltration.

Nevertheless, our results suggest that the combination treatment mechanism is dependent upon CD4+ non-Treg cells and may have some dependence upon CD8+ cells (Fig. 2). While the treatment mechanism was entirely abolished after depletion of CD4+ cells, anti-GITR (1)/SRS treatment after CD8+ cell depletion did not produce long-term survivors, but also did not significantly reduce median survival compared to non-depleted mice (Fig. 2a-b). Our results align with previous studies of TNFR checkpoint stimulation in combination with SRS in intracranial glioma, which indicated that CD4+ depletion abrogated treatment effect while CD8+ depletion did not [5]. While studies of anti-GITR in flank melanoma models have demonstrated CD8+ mechanistic dominance, more recent studies in flank tumor have emphasized the importance of CD4+ helper-T cells in coordinating a CD8+ anti-tumor response [12, 13, 36]. The difference in T cell subset dominance between studies in melanoma and our findings may be rooted in the immunologic distinctiveness of the tumor microenvironment in flank *versus* intracranial tumor models [27, 37].

In support of Th1-type CD4+ T cell involvement in our combination treatment mechanism, we observed a significantly elevated CD4 + IFNγ + to Treg ratio in our combination treatment group, as well as elevated CD4+ production of IFNγ and IL-2 and CD8+ production of IFNγ and TNFα (Fig. 3). Corroborating our observations in Fig. 2, while the CD8 + IFNγ + to Treg ratio was elevated in our combination treatment relative to control, the difference was not statistically significant (Fig. 3c). Together, these data suggest a possible involvement of CD8+ T cells in the anti-tumor response. While our results supported previous findings of the increase in intratumoral multifunctional CD8+ T cells after GITR stimulation, others observed significantly elevated CD8+ effector to Treg ratios and direct co-stimulatory effects on CD8+ cells [12, 13, 38]. Further investigation in the intracranial glioma model is necessary to more definitively ascertain the role of CD8+ cells in the anti-GITR (1)/SRS treatment effect. Moreover, of importance for future study is the combination of SRS with Treg depletion. Our results demonstrated elevated Treg levels in the presence of SRS alone (Fig. 1e), as well as mildly elevated IFNγ + effector T cells (Fig. 3). Future investigation may involve augmentation of anti-tumor effect with the combination of focal radiation and Treg depletion.

As CD4+ effector cells are not commonly the cytotoxic effector cells in an immune response, we hypothesized that the combination treatment induced M1 polarization of mononuclear cells in the tumor microenvironment, potentially recruited by IFNγ-secreting CD4+ cells. Macrophages may be roughly categorized as either M1 or M2 based on their overall gene expression pattern, but this distinction is not absolute as macrophages may lie on a phenotypic spectrum [25]. Macrophages that are M1 are ‘classically activated’ and anti-tumorigenic, whereas M2 macrophages are ‘alternatively activated,’ pro-tumorigenic, and are associated with poor immune responses. With the exception of *Inos*, we observed significantly elevated expression of select stereotypically M1 genes and decreased expression of M2 genes in intratumoral CD11b + CD45+ mononuclear cells in the combination treatment group, as well as decreased expression of *Pdl1* and *Tgfb* (Fig. 4). Cytokines released by local T cells are known to influence macrophage polarization, with elevated IFNγ release by Th1 cells promoting an M1 phenotype [25, 39]. Indeed, our results indicate a significantly increased proportion of CD4 + IFNγ + cells in the presence of anti-GITR (1)/SRS treatment, which may in turn favor macrophage M1 polarization. We predict that CD4+ Th1 cells may be dominant in the anti-GITR (1)/SRS treatment mechanism because of their integral role in macrophage polarization toward an M1 phenotype in the tumor microenvironment. A previous study in murine ovarian cancer treated with PD-1 blockade combined with GITR stimulation showed a significant decline in myeloid derived suppressor cells (MDSCs) [16]. Our results corroborate the observation of a decline in suppressive myeloid type cells after anti-GITR treatment.

Finally, we present novel data that anti-GITR IgG2a mAb alone or in combination with SRS does not mediate a survival advantage and is not capable of depleting Tregs in intracranial tumor (Fig. 5a–c). Anti-GITR (2a) relies upon activating FcγR engagement to mediate cell death of GITR-expressing cells via ADCC [26]. Because regulatory T cells express constitutively high levels of GITR, they are the primary cell population targeted for cell death in the presence of anti-GITR (2a). It is important to note that in order for ADCC to occur, antigen presenting cells (APCs) must be present that express the appropriate Fc receptor specific to the antibody of interest. Systemic tumor models treated with anti-GITR (2a) undergo significant regression as well as depletion of Tregs in a mechanism dependent upon activating FcγR, including receptor variants III and IV [26]. We illustrate that the primary APCs of the brain, the microglia, express significantly decreased levels of FcγRIII and IV relative to macrophages infiltrating from the periphery (Fig. 5d). Moreover, anti-GITR (2a) treatment resulted in significant Treg depletion in flank GL261 tumors, suggesting that factors unique to the intracranial compartment are responsible for the lack of anti-GITR (2a) mediated Treg depletion in brain tumor. These data may provide a partial explanation for the lack of tumoral Treg depletion and absence of survival benefit in our intracranial glioma model after treatment with anti-GITR (2a)/SRS, despite previous observations of systemic tumor regression after anti-GITR (2a) treatment [26]. We assume that microglia are the primary professional APCs of the CNS, and that their expression of FcγR would be of greatest importance in evaluating the effect of anti-GITR (2a)-mediated ADCC. While infiltrating peripheral mononuclear cells may play a role in FcγR-mediated ADCC in the presence of anti-GITR (2a), this question is multidimensional and requires further investigation. Namely, in addition to an enumeration of intratumoral infiltrating mononuclear cells, the timing of infiltration relative to antibody administration, as well as the spatial distribution within the tumor architecture is of great importance.

Nevertheless, our findings imply that depleting antibodies dependent upon FcγR interactions, such as anti-CTLA-4 or anti-OX40, may not be of clinical value in brain cancer if the appropriate FcγR are also absent from human microglia [40, 41]. Additional studies in humans are necessary to confirm this hypothesis.

## Conclusions

Together, these preclinical findings provide a strong rationale for the clinical testing of anti-GITR (1) and adjuvant focal radiation combination therapy in the setting of human GBM. The clinical translation of these therapies to the clinic would be facile, given that a human anti-GITR antibody is available (MK-4166) and SRS is part of GBM standard of care. Data regarding immunologic mechanisms we have presented here may serve as a context for investigating biomarkers in future clinical trials.

## Methods

### Mice, reagents, and antibodies

Female C57BL/6 mice (The Jackson Laboratory) and FoxP3^DTR^ C57BL/6 (provided by Alexander Rudensky at Memorial Sloan Kettering Cancer Center) mice (6–8 weeks) were housed in facilities in accordance with protocols approved by the Institutional Animal Care and Use Committee of Johns Hopkins University. Anti-GITR IgG1 D265A and IgG2a (Clone mGITR.7-mg2a) antibodies were provided by Bristol-Myers Squibb Company, and purified anti-CD4 (Clone GK1.5) and anti-CD8 (Clone 53–6.7) antibodies were purchased from BioXcell (Cat. BE0003-1 and BE0004-1, respectively). Anti-GITR, anti-CD4, and anti-CD8 antibodies were all diluted to 1 mg/kg and stored at 4 °C. Diphtheria toxin was purchased from Sigma (Cat. D0564-1MG), diluted to a concentration of 5 μg/mL, and stored in 1 mL single-use aliquots at−80 °C. GL261-luc is a mouse-derived glioma cell line purchased from Caliper, and grown in DMEM (Thermo Fisher Scientific) supplemented with 10 % fetal bovine serum, 100 units/mL penicillin, 100 μg/mL streptomycin, and 100 μg/mL G418 for selection. This cell line was confirmed to be mycoplasma free using the MycoDtect kit (Greiner Bio-One) performed at the Fragment Analysis Facility at our institution.

### Tumor establishment and antibody treatment

GL261-luc cells were maintained in cell culture in selection media. Cells were grown to log phase, harvested, washed thoroughly three times in PBS, counted, and brought to the appropriate concentration in PBS. Cells were resuspended at 130,000 cells/μL for intracranial implantation and at 20,000 cells/μL for flank implantation.

Establishment of intracranial tumors was achieved as previously described [6]. Briefly, mice were anesthetized with 200 μl of ketamine (5 mg/mL)/xylazine (0.5 mg/mL) in PBS and their heads were shaved, cleaned with povidone-iodine, and incised at midline. A burr hole 1 mm in diameter was placed over the left hemisphere 2 mm posterior to the coronal suture and 2 mm lateral to the sagittal suture. After positioning mice in a stereotactic frame, the needle was advanced 3 mm below the dura. Tumor cells were injected in a volume of 1 μL over 1 min. The skin was closed with staples. Flank tumors were established by subcutaneous injection of the tumor cell suspension in a volume of 100 μL into the left flank. Intracranial and flank tumor establishment was confirmed by tumor bioluminescent imaging.

Anti-GITR antibodies were dosed at 10 mg/kg in a volume of 200 μL by intraperitoneal (i.p.) injection 10, 13, and 16 days after tumor implantation. It was confirmed that administration of an anti-GITR matched isotype control antibody led to no appreciable differences in result when compared to the lack of antibody administration in control and SRS treatment only mice.

### Radiation therapy

Focal radiation was delivered in one fraction on day 10 after tumor implantation using the small animal radiation research platform [42] commercialized as SARRP (Xstrahl). Radiation was delivered in a 3 mm vertical beam at a rate of 1.9 Gy/min to 10 Gy centered over the tumor with CT guidance as previously described [6].

### Survival experiments and bioluminescent imaging

After intracranial implantation of 130,000 GL261-luc cells into the left hemisphere, mice were treated with anti-GITR mAb (10 mg/kg) and SRS (10 Gy) as described above. Tumor burden was monitored by luciferase imaging using an IVIS Spectrum In Vivo Imager (Caliper) as previously described [6]. Mice were sacrificed according to protocol when they developed a hunched posture or ambulatory deficits impairing feeding behavior.

### Flow cytometry

An LSR II (BD Biosciences) was used for flow cytometry. The following antibodies were used: LIVE/DEAD Aqua (Life Technologies, L34957), CD3 APC/Cy7 (BioLegend, 300317), CD3e PerCP/Cy5.5 (BD, 561108), CD4 PB (Invitrogen, MHCD0428), CD8 BV605 (BD, 564115), CD25 PE-Cy7 (eBioscience, 25025942), Foxp3 PE (eBioscience, 12477182), IFN-γ PE-Cy7 (eBioscience, 25731141), TNF-α FITC (BD, 554418), IL-2 APC (BD, 562041), F4/80 PE-Cy7 (BioLegend, 123113), CD16/CD32 APC (eBioscience, 17016181), CD16-2/FCGR4 PE (SinoBiological, 50036R012P10), CD11b FITC (eBiosciences, 11011281), CD45 AF700 (BioLegend, 103127).

### In vivo T cell subtype depletion

Depletion of CD4+ and CD8+ cells was achieved by i.p. injection of 10 mg/kg of GK1.5 or 53–6.7 antibody in a volume of 200 μL on days 5–7, 14, and 21 after tumor implantation. Depletion of FoxP3+ cells in FoxP3^DTR^ mice was achieved by i.p. injection of 50 ng/g of diphtheria toxin in a volume of 200 μl on day 8 and 9 after tumor implantation and every 2 days thereafter until euthanasia to maintain depletion. Depletion of >99 % of T cell subsets was confirmed by testing peripheral blood of control mice for the presence of CD4+, CD8+, or Foxp3+ cells using flow cytometry.

### Immunophenotyping of tumor-infiltrating lymphocytes and myeloid cells

For intracranial tumors 130,000 GL261-luc cells were implanted in the left hemisphere, and for flank tumors 2 million GL261-luc cells were implanted subcutaneously in the left flank. Mice were treated with anti-GITR antibody on days 10, 13, and 16 after implantation and with SRS on day 10 after implantation. Mice with intracranial tumors were sacrificed on day 21 post implantation and with flank tumors were sacrificed on day 18 post implantation. Tumors were excised from surrounding tissue, homogenized, and infiltrating lymphocytes or mononuclear cells were isolated using centrifugation on a Percoll (Sigma) density gradient. For immune cell isolation, a working solution of Percoll was prepared by combining 90 % Percoll and 10 % HBSS (10×), which was then used to produce 40 and 80 % solutions for lymphocyte isolation and 30 and 70 % solutions for mononuclear cell isolation. Gradients were spun for 20 min at 2000 RPM at room temperature with no brake. The resultant cell layer was collected from the density interface and thoroughly washed in 50 mL of PBS. Red blood cells were lysed from harvested spleen samples. Tumor draining lymph nodes (TDLN), considered deep cervical lymph nodes for intracranial tumors and inguinal lymph nodes for flank tumors, were carefully dissected, homogenized, and washed in 1 mL of PBS. When indicated, harvested lymphocytes from tumor, spleen, and TDLN were stimulated with phorbal 12-myristate 13-acetate (PMA)/Ionomycin in the presence of Golgi Stop for 4 h at 37 °C. Cells were then washed and stained for appropriate intracellular or extracellular markers and analyzed by flow cytometry on an LSR II (BD).

### Quantitative RT-PCR

Mice were implanted intracranially with 130,000 GL261 cells in the left hemisphere, treated with anti-GITR antibody on days 10, 13 and 16 and with SRS on day 10 post implantation. Mice were sacrificed on day 21 after implantation. Tumors were excised, processed, and mononuclear cells isolated with centrifugation on a density gradient (Percoll). Resulting cells were washed, stained for CD11b and CD45, and sorted for CD11b + CD45+ cells on a FACSAria II (BD) into TRIzol Reagent (Life Technologies) to extract mRNA using the TRIzol RNA Isolation Protocol. Cellular mRNA was quantified using a Nanodrop 8000 UV spectrophotometer. RNA (1 μg) was converted to cDNA using the RNA to cDNA EcoDry Premix (Clontech). Resultant cDNA was used as a template to target mouse *IL12* (p35 subunit transcript)*, IL10, Tgfb, MhcII, Cd86, Pdl1, Cd163, Cd206, Arg1,* and *Inos*. Primers were purchased from Life Technologies-Applied Biosystems. An Applied Biosystems StepOnePlus instrument was used to amplify samples in triplicate. The ΔΔC_t_ method was used to calculate quantity of mRNA expression relative to the average of the control treatment group.

### Statistics

Data were analyzed with log-rank test and unpaired student’s *t* test on GraphPad Prism software. Significant *p*-values were those less than 0.05. Experiments were repeated *x*2–3.

## Additional file

Additional file 1: Figure S1. Isolated CD45 + CD11b + tumor infiltrating mononuclear cells. C57BL/6 mice were inoculated with GL261-luc tumor, randomized to groups of ≥5, and dosed with anti-GITR (1) and SRS as in Fig. 1. Mice were sacrificed on day 21, tumor infiltrating mononuclear cells were isolated, cells were stained with extracellular markers and sorted by the indicated CD45 + CD11b + gate. Numbers within boxes indicate gated percentage of total mononuclear cell population; numbers below boxes indicate absolute cell count of gated cells. (PDF 60 kb)
