# Erratum to: Agonist anti-GITR monoclonal antibody and stereotactic radiation induce immune-mediated survival advantage in murine intracranial glioma

**DOI:** 10.1186/s40425-016-0181-6

**Published:** 2016-11-04

**Authors:** Mira A. Patel, Jennifer E. Kim, Debebe Theodros, Ada Tam, Esteban Velarde, Christina M. Kochel, Brian Francica, Thomas R. Nirschl, Ali Ghasemzadeh, Dimitrios Mathios, Sarah Harris-Bookman, Christopher C. Jackson, Christina Jackson, Xiaobu Ye, Phuoc T. Tran, Betty Tyler, Vladimir Coric, Mark Selby, Henry Brem, Charles G. Drake, Drew M. Pardoll, Michael Lim

**Affiliations:** 1 The Johns Hopkins University School of Medicine, Baltimore, USA; 2 Department of Oncology, Baltimore, USA; 3 Department Radiation Oncology, Baltimore, USA; 4 Department of Neurosurgery, The Johns Hopkins University School of Medicine, 600 N. Wolfe St. Phipps Building Rm 123, Baltimore, 21287 MD USA; 5 Bristol-Myers Squibb Company, San Francisco, CA USA; 6 The Brady Urological Institute, Baltimore, USA

## Erratum

Unfortunately, after publication of this article [1], it was noticed that a funding source was not mentioned. Bristol-Myers Squibb was intended to be included in the ‘Financial Support’ section of the article.

## References

[1] Patel MA, Kim JE, Theodros D, Tam A, Velarde E, Kochel CM, Francica B, Nirschl TR, Ghasemzadeh A, Mathios D, Harris-Bookman S, Jackson CC, Jackson C, Ye X, Tran PT, Tyler B, Coric V, Selby M, Brem H, Drake CG, Pardoll DM, Lim M (2016). Agonist anti-GITR monoclonal antibody and stereotactic radiation induce immune-mediated survival advantage in murine intracranial glioma. J Immunothera Cancer.

