# Supplementary figures and images for: Agonist anti-GITR monoclonal antibody and stereotactic radiation induce immune-mediated survival advantage in murine intracranial glioma
Source: J Immunother Cancer. 2016 May 17;4:28. doi: 10.1186/s40425-016-0132-2 (PMC4869343; doi:10.1186/s40425-016-0132-2)

CD45

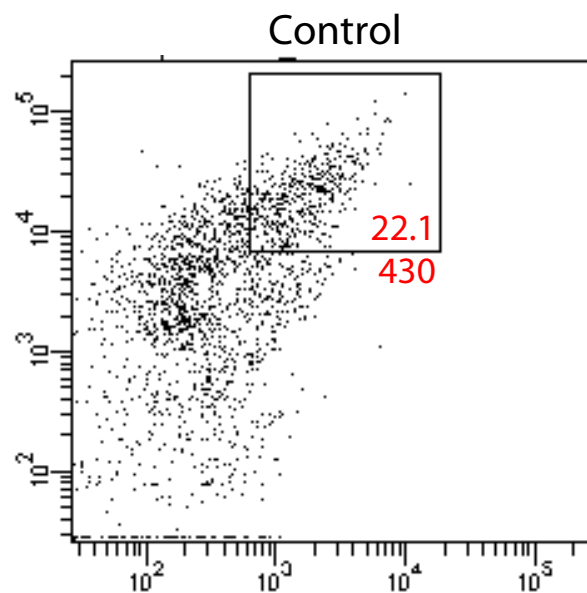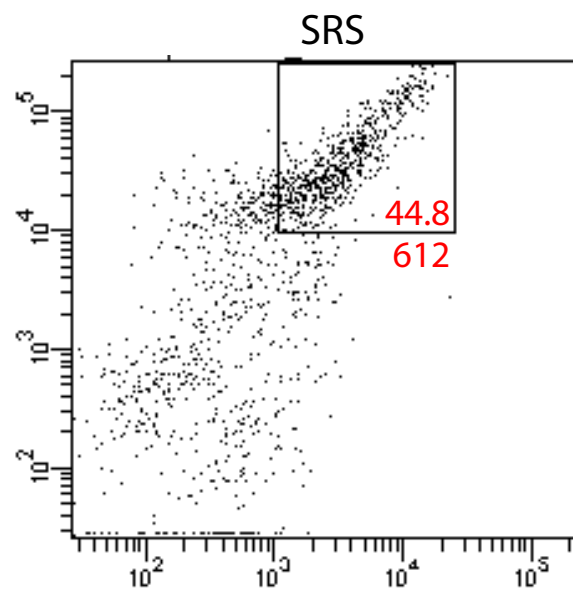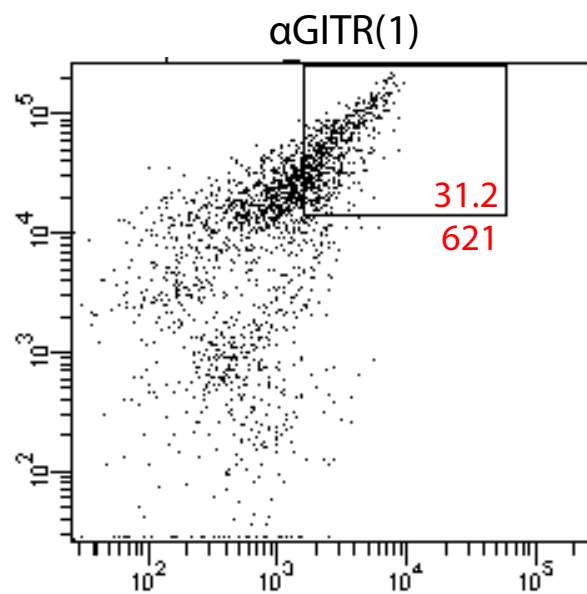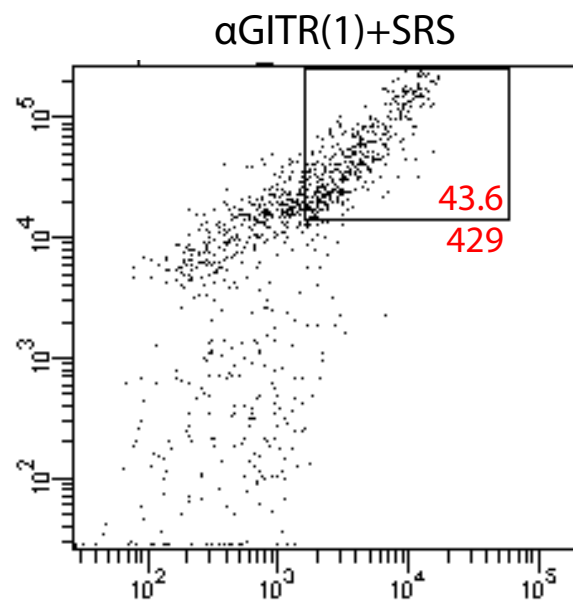

CD11b

Supplement: Additional file 1: Figure S1. — Isolated CD45 + CD11b + tumor infiltrating mononuclear cells. C57BL/6 mice were inoculated with GL261-luc tumor, randomized to groups of ≥5, and dosed with anti-GITR (1) and SRS as in Fig. 1. Mice were sacrificed on day 21, tumor infiltrating mononuclear cells were isolated, cells were stained with extracellular markers and sorted by the indicated CD45 + CD11b + gate. Numbers within boxes indicate gated percentage of total mononuclear cell population; numbers below boxes indicate absolute cell count of gated cells. (PDF 60 kb) [file 40425_2016_132_MOESM1_ESM.pdf]
